# The Importance of Inflammatory and Angiogenic Markers in the Evaluation of Early Cardiovascular Disease Risk in Women with Hypertensive Disorders of Pregnancy

**DOI:** 10.3390/jcdd10100407

**Published:** 2023-09-22

**Authors:** Tatjana Maselienė, Emilija Struckutė, Rūta Breivienė, Diana Ramašauskaitė, Vilma Dženkevičiūtė

**Affiliations:** 1 Clinics of Internal and Family Medicine, Faculty of Medicine, Vilnius University, LT-01513 Vilnius, Lithuania; 2 Clinics of Obstetrics and Gyneacology, Faculty of Medicine, Vilnius University, LT-01513 Vilnius, Lithuania; emilija.struckute@stud.mf.vu.lt (E.S.); ruta.einikyte@santa.lt (R.B.); diana.ramasauskaite@santa.lt (D.R.); 3 Clinics of Cardiology, Faculty of Medicine, Vilnius University, LT-01513 Vilnius, Lithuania; vilma.dzenkeviciute@santa.lt

**Keywords:** hypertensive disorders of pregnancy, preeclampsia, angiogenic factors, placental growth factor, soluble fms-like tyrosine kinase-1, interleukin-6, tumor necrosis factor alpha, arterial hypertension

## Abstract

Background: Women with hypertensive disorders of pregnancy (HDP) have a significantly higher risk of developing cardiovascular diseases later in life. The stratification of this risk using biomarkers during pregnancy can help to identify these women and apply early prevention. Objective: We aimed to determine proinflammatory cytokines and angiogenic markers, echocardiographic parameter changes after delivery and predict early cardiovascular disease risk in women with arterial hypertension and its complications during pregnancy. Methods: We conducted a literature search using the PubMed database for the last ten years. A total of 17 articles were included to our study and full text reviewed. Results: Four out of six studies found higher postpartum Interleukin-6 (IL-6) levels in women with HDP. IL–6 correlated positively with waist circumference, body mass index, and triglycerides, and negatively with high density lipoproteins (HDL). Two out of four studies found higher postpartum tumor necrosis factor alpha (TNF-α) levels in women with HDP but later concentration equalizes. One out of eight studies found higher placental growth factor (PlGF) and two out of eight found more elevated soluble fms-like tyrosine kinase-1 (sFlt-1) in women with HDP. With decreasing PlGF and increasing sFlt-1, common carotid artery intima and media thickness, aortic root diameter, left atrial diameter, left ventricle mass, systolic, diastolic, and mean blood pressure increased, whereas HDL decreased. One out of four studies found higher sFlt-1/PlGF. Conclusion: IL-6 remains significantly higher after delivery. Few studies found higher TNF-α, sFlt-1, PlGF and their ratio postpartum. All studies found a correlation between angiogenic factors, IL-6, and cardiovascular disease risk factors.

## 1. Introduction

Hypertensive disorders are the main cause of morbidity and mortality among women during pregnancy [1]. Hypertensive disorders of pregnancy (HDP) are chronic hypertension, gestational hypertension, preeclampsia (PE), and eclampsia, which occur in 5–10% of all pregnancies [2]. Based on statistical medical data from the Lithuanian Institute of Hygiene, it has been found that 5.2% of pregnant women in Lithuania experienced hypertensive disorders during pregnancy in 2021. Ranging in severity, hypertensive disorders of pregnancy include chronic hypertension (systolic blood pressure (BP) ≥ 140 mmHg or diastolic BP ≥ 90 mmHg that predates the onset of pregnancy); gestational hypertension (hypertension diagnosed after 20-week gestation without concurrent proteinuria); preeclampsia–eclampsia (classically, new-onset hypertension with new-onset proteinuria); and chronic hypertension with superimposed preeclampsia (chronic hypertension with new-onset proteinuria or other signs/symptoms of preeclampsia after 20-weeks or chronic proteinuria with new-onset hypertension) [3].

Preeclampsia is a pregnancy disorder characterized by hypertension and systemic endothelial impairment. Globally, PE complicates about 2–8% of all pregnancies [4]. Delivery usually resolves all PE symptoms, but sometimes, PE can start de novo in the postpartum period [3]. 

Hypertensive disorders of pregnancy are also associated with a higher risk of cardiovascular diseases in the long term [5]. Preeclampsia is associated with a 4.19-fold risk of developing heart failure, 2.5-fold risk of developing coronary heart disease, 1.81-fold risk of stroke, and overall a 2.21-fold risk of death because of cardiovascular diseases (CVD) [6]. However, there is a lack of recommendations and prevention programs for CVD in the postpartum period. Early risk assessment and targeted treatment might prevent future CVD. Studies have shown that women with HDP have different risks of CVD later in life. Compared to late-onset PE, early-onset PE is associated with a higher risk of cardiovascular and cerebrovascular events, hypertension, dyslipidemia, and metabolic syndrome [7]. Furthermore, there is a greater risk of cardiovascular disease (CVD) in women with gestational hypertension (GH) who have both preterm delivery and small for gestational age infants, compared to those with GH alone. [8]. Recurrent preeclampsia is associated with an increased risk of hypertension, ischemic heart disease, heart failure, cerebrovascular accident, and hospitalization due to CVD compared with preeclampsia in a single pregnancy followed by normal pregnancy [9]. Studies about traditional modifiable CVD risk biomarkers are non-homogenous as some of them indicate higher rates of glucose, body mass index (BMI), high density lipoproteins (HDL), low density lipoproteins (LDL), triglycerides in women with HDP. In contrast, others do not find significant differences in these biomarkers between women with HDP and normal pregnancies, postpartum or during pregnancy [10,11,12,13,14]. Using novel biomarkers could help to identify women with a greater risk of CVD. 

The mechanism of increased CVD risk is unclear, but there are two possible pathways. The first one is that women with HDP have an increased CVD risk profile before pregnancy as HDP and CVD share some of the same risk factors such as obesity, smoking, alcohol intake, age, diabetes, and family history. The HDP might be the first manifestation of increased CVD risk [15]. The other one is that preeclampsia generates a long-lasting endothelial and cardiac dysfunction contributing to further CVD development [16]. Preeclampsia is a state of chronic inflammation with an immune imbalance where proinflammatory cytokines are increased (TNF-α, IL-6, IL-17) and anti-inflammatory cytokines are reduced (IL-10, IL-4) [17]. TNF-α and IL-6 increase gradually with the severity of preeclampsia from gestational hypertension to mild preeclampsia and severe preeclampsia [18]. Preeclampsia is also associated with placental malperfusion because of inadequate spiral arteries remodeling. Placental hypoxia results in syncytiotrophoblasts dysfunction. In response to hypoxia, syncytiotrophoblasts decrease the production of PlGF and increase the production of sFlt-1. The imbalance of angiogenic factors results in endothelial dysfunction [19]. As these angiogenic and inflammatory factors are involved in the pathogenesis of HDP, they might be useful in early CVD risk stratification for women with HDP and potential therapeutical targets. This narrative review aimed to examine the current knowledge about the association between inflammatory, angiogenic factors and early cardiovascular disease risk. 

## 2. Materials and Methods

The literature search was performed using the PubMed database and included articles since 1 November 2004. The PubMed database was used because of free access from our university. The time frame was 19 years. Arterial hypertension and its complications during pregnancy was the exact object of analysis. After reviewing the database, we selected those pro-inflammatory and angiogenic markers that have been studied the most during pregnancy. Used keywords were placental growth factor, soluble fms—like tyrosine kinase-1, interleukin-6, interleukin-10, tumor necrosis factor alpha, cardiovascular disease risk, hypertensive disorders of pregnancy, and preeclampsia. The database generated 2598 articles. Initial screening was conducted on the basis of the article’s title and abstract screen, followed by full manuscript review in duplicate for articles that appeared to be relevant. Consensus was achieved by three reviewers (E.S., T.M., V.D.) in the event of uncertainty or disagreement. We identified 25 articles to investigate further. Once again, two reviewers (T.M. and E.S.) thoroughly reviewed the full text of the articles and finally included 12 articles in our research. The article selection was performed from December 2021 to February 2022. Unfortunately, we had to exclude 13 articles. We did not include reports that were prospective studies or literature reviews or focused on HDP and other health conditions. We also excluded articles that only measured biomarkers during pregnancy or studied biomarkers triggered by inflammation. If we could not access the full-text version of the article, we did not include it. Ultimately, we added five articles to the list from references and had all 17 articles in our study. 

## 3. Results

All results are listed below in Table 1. 

### 3.1. TNF-α

Preeclampsia is associated with significantly higher levels of the proinflammatory cytokine TNF-α than uncomplicated pregnancies [31]. TNF-α is produced by the placenta and innate immune cells [32]. It is a pro-atherogenic cytokine that promotes atherosclerosis and activates leukocyte adhesion to the endothelium [33]. TNF-α increases during all pregnancy and has been related to miscarriages, fetal losses, preeclampsia, and preterm birth and is associated with IL-10 reduction [34]. There seemed to be an essential antagonistic balance between TNF-α and IL-10 ratio during implantation and pregnancy. Both cytokines were produced by immune cells and trophoblast. An increase in the TNF-α/IL-10 ratio potentially causes early and late pregnancy complications, mostly implantation failure, fetal loss, hypertensive syndromes, and gestational diabetes [35,36,37]. In four case–control studies, researchers examined postpartum TNF-α changes in plasma. Two out of four studies found significantly higher levels of TNF-α in women with a history of HDP compared to uncomplicated pregnancies [11,22]. Preeclamptic women 12–14 weeks postpartum had significantly higher levels of TNF-α than those with normotensive pregnancy [22]. Another study found significantly higher TNF-α in pregnancies complicated by hypertensive disorders one year postpartum [11]. Two studies showed no significant differences between case–control TNF-α plasma levels measured 3, 9 months, and 9–16 years postpartum [10,26]. The inconsistency among the studies might be due to the fact that TNF alpha levels decrease over time in the postpartum period. One study grouped patients with a history of HDP together with women who had preterm and small-for-gestational-age newborns. Additionally, there was a small number of participants. 

### 3.2. IL-6

IL-6 is a proinflammatory cytokine secreted excessively by maternal immune cells in preeclampsia [38]. The IL-6 and TNF-α cytokines increase vascular permeability and induce apoptosis of trophoblastic cells [39]. IL-6 also acts as a pro-fibrotic factor and stimulates cardiac fibrosis [40]. Chronic elevation of IL-6 induces myocardial hypertrophy and decreases contractility [41]. IL-6 is expressed in carotid atherosclerotic plaques. Also, circulating levels of IL-6 are higher in patients with atherosclerotic plaques than those without [42]. Of the six case–control studies, four found elevated postpartum IL-6 levels in pregnancies complicated by hypertensive disorders. Significantly higher IL-6 levels were discovered 12–14 weeks after delivery in preeclamptic women than in controls [22]. IL-6 levels were higher among women with cardiovascular disease related pregnancy complications (hypertensive disorder of pregnancy, preterm birth, and small for gestational age baby) 3 and 9 months postpartum [10]. A total of 9–16 years postpartum plasma level of IL-6 was higher in the early-onset preeclampsia group than in the uncomplicated pregnancy group [26]. IL-6 levels were also higher among women with a history of HDP than normotensive pregnancies 12–22 years postpartum [29]. Another two studies did not find any significant difference in IL-6 in case–control groups one year and twenty years postpartum [11,30] but found elevated IL-6/IL-10 ratio 20 years postpartum in the HDP group [30]. Researchers found that IL-6 measured 9–16 years postpartum in women with a history of early-onset preeclampsia relates to metabolic syndrome features such as waist circumference, BMI, fasting triglycerides, and fasting HDL cholesterol [26]. 

### 3.3. Angiogenic Factors

Placental growth factor (PlGF) is a proangiogenic factor secreted mostly by the placenta. The main function of PlGF is placental vascularization. During HDP, the secretion of PlGF decreases, resulting in hypertension. Soluble fms-like tyrosine kinase-1 (sFlt-1) is an anti-angiogenic factor that binds to PlGF and reduces availability. Decreased PlGF and increased sFlt-1 result in endothelial dysfunction and impaired hemodynamics [16]. In two of eight case–control studies, researchers found higher postpartum sFlt-1 levels in women with a history of HDP compared to the control group. One year postpartum, sFlt-1 decreased in both preeclampsia and control groups but remained significantly higher in the preeclampsia group. Also, women with PE had a higher sFlt-1/PlGF ratio [24]. Five to eight years postpartum, sFlt-1 levels were higher in the PE group before and after adjustment for BMI, but p-value was marginal, equal to 0.05 [25].Only one study of eight found higher PlGF serum levels three months postpartum in women with a history of HDP [23]. Decreased PlGF levels are associated with higher systolic, and diastolic, mean blood pressure and lower HDL [14,28]. Higher sFlt-1 levels measured in preeclamptic women during their pregnancy correlate with higher mean arterial pressure 12 years postpartum, but there was no correlation between sFlt-1 and BMI [43]. The higher sFlt-1 and sFlt-1/PlGF were during pregnancy, the lower HDL was 12 years postpartum [28].

### 3.4. Traditional Cardiovascular Risk Factors

Findings about traditional cardiovascular risk factors in preeclamptic patients are nonhomogenous. Researchers found that HDL, LDL, total cholesterol, TAG and calprotectin did not differ in PE group compared to control group, but high sensitivity C-reactive protein, mean arterial pressure, systolic blood pressure were found to be higher in PE patients 5–8 years postpartum [25]. At 12 years postpartum, PE patients had more cases of dyslipidemia and arterial hypertension compared to control patients, but there were no significant difference in body mass index (BMI), smoking status, metabolic syndrome, glucose, LDL, total cholesterol, and TAG [28]. 

### 3.5. Arterial Aging Signs and Angiogenic Factors

The extent of atherosclerotic plaques can be evaluated by measuring common carotid artery intima-media thickness (cIMT). This test works as a subclinical marker of atherosclerosis progression. Thicker intima, thinner media, and a higher intima-media ratio (I/M) were found in women with preeclampsia compared to normal pregnancies during diagnosis [24]. sFlt-1 and sFlt-1/PlGF correlated positively and PlGF negatively with intima thickness and intima-media ratio during diagnosis and one year postpartum [24]. After adjusting for BMI, blood pressure, smoking status, and family history of CVD, the correlation between angiogenic factors and arterial wall measurements remained significant [24]. The same research found that higher sFlt-1 and sFlt-1/PlGF ratio were associated with higher BMI and blood pressure [24]. Angiogenic factors measured during pregnancy correlated with cIMT 12 years postpartum, sFlt-1 correlated positively, and PlGF negatively [28]. Also, cIMT was higher in the preeclampsia group than in controls 12 years postpartum [28]. All changes are listed below in Table 2.

### 3.6. Angiogenic Factors and Echocardiogram Measurements

Left ventricle global longitudinal strain (LV GLS) measures left ventricle systolic function indicating subclinical left ventricle dysfunction [44]. LV GLS indicates the maximal shortening of myocardial longitudinal length during systole compared to the resting length in diastole [45]. GLS was worse in women with preeclampsia than those with chronic or gestational hypertension and those with normotensive pregnancy. Also, sFlt-1 levels correlated with worse GLS at the time of diagnosis of preeclampsia [43]. Higher sFlt-1 levels were measured when severe preeclampsia was diagnosed and were associated with worse LV GLS 4–12 weeks postpartum [21]. PlGF during pregnancy correlated negatively with GLS. sFlt-1 correlates positively with left ventricular posterior wall thickness [28]. Elevated sFlt-1 levels detected during pregnancy were associated with increased chamber and vascular stiffness measurements (such as end systolic elastance, and arterial elastance) during pregnancy and 4–12 weeks postpartum. Higher sFlt-1 levels were associated with the worse diastolic function (higher E/E’ at the time of diagnosis, decreased E/A at the time of diagnosis, and 4–12 weeks postpartum) [21]. Laura Benschop et al., found that individuals with lower mid-pregnancy PlGF levels had larger aortic root diameter, left atrial diameter, and left ventricular mass six years postpartum. The study included women with a history of HDP and individuals from all populations. Results persisted the same after excluding women with complicated pregnancies [14]. Increased levels of sFlt-1 measured during pregnancy correlated with increased left ventricular mass, but there was no correlation with ejection fraction [43]. All changes are listed below in Table 3 and Table 4.

## 4. Discussion

Hypertensive disorders of pregnancy are associated with increased cardiovascular disease (CVD) risk later in life. The American Heart Association identifies preeclampsia and gestational hypertension as CVD risk factors [46]. The diagnosis of HDP might be an opportunity for risk stratification and targeted prevention of CVD, but the risk stratification and treatment methods currently need to be improved. The researchers found that angiogenic factors measured during the pregnancy correlate with CVD risk factors (blood pressure, arterial aging signs, HDL, cardiac remodeling) evaluated postpartum. Also, in most studies, authors found no significant difference between women with a history of HDP and normal pregnancies in the postpartum levels of angiogenic factors. During pregnancy, it is important to measure angiogenic factors not only to predict preeclampsia but also to stratify the risk of cardiovascular disease. As angiogenic factors equalize after pregnancy between women with HDP and normal pregnancies, they might not contribute to postpartum CVD development.

It is necessary to include more details regarding specific levels of angiogenic factors and how they relate to the risk of cardiovascular disease. Laura Benschop et al., in their study, divided PlGF into quartiles and found that the lowest quartile had the highest chance of being evaluated by blood pressure and echocardiography [14]. Also, the best time for biomarkers measurement during pregnancy is unknown. However, Rugina I. Neuman et al., found that sFlt-1, PlGF, and their ratio cannot predict hypertension one year postpartum [47].

The relationship between biomarkers and subsequent cardiovascular events and mortality in women with a history of HDP is unclear. However, increased levels of sFlt-1 and decreased PlGF are found in heart failure, and they are significantly related to New York Heart Association (NYHA) classes (*p* < 0.001) [48]. Higher sFlt-1 levels are associated with atherosclerotic cardiovascular disease events (cardiovascular death, myocardial infarction, stroke, revascularization) [49]. It was shown that patients with acute decompensated heart failure and higher PlGF had worse prognosis than the low PlGF patients in terms of all-cause and cardiovascular death [50]. Anti-TNF-α therapy in rheumatoid arthritis patients reduces the risk of all cardiovascular events (myocardial infarction, cerebrovascular accident, congestive heart failure) [51]. Patients with heart failure, who had higher levels of TNF-α, were found to have a lower survival rate [32]. Acute coronary syndrome patients in the highest quartile of IL-6 have a higher risk of cardiovascular events and death [52]. To our best knowledge, there are no studies between biomarkers and future cardiovascular events and mortality in women with history of HDP.

Discussed biomarkers might give an additional value when evaluating cardiovascular events risk, not only in pregnant women. The use of both BNP and PlGF improves prediction of cardiovascular events in chronic kidney disease patients compared to BNP alone [53]. High placental growth factor levels can indicate increased cardiac risk in patients with undetectable, low, and high troponin T levels [54]. sFlt-1 and PlGF correlate with cardiovascular risk factors such as galectin-3, PTH, and aldosterone, sFlt-1 correlates with NT-proBNP [48]. Other researchers found correlation between sFlt-1 and D-dimers, but not with NT-proBNP [49]. BNP and troponin I were shown to exhibit the strongest association and sFlt-1 exhibited the next strongest association in a study which evaluated relationship between 9 biomarkers and hospitalization in heart failure patients [55]. The biomarkers discussed in our article could be an excellent indicator for further cardiovascular risk assessment, but additional studies are needed.

Echocardiographic changes can also be used to predict cardiovascular diseases. Cardiac function and morphology do not change immediately before the delivery and early postpartum period [56]. Victoria A. deMartelly et al., found that GLS, left ventricular posterior wall thickness, and interventricular septal thickness stay elevated ten years postpartum in preeclamptic women [57]. Chahinda Ghossein-Doha et al., found that chronic hypertension diagnosed six years postpartum was associated with increased left ventricular mass index and diastolic blood pressure at nine months postpartum [58]. 

Our exploration indicates that women with a history of HDP have higher levels of IL-6 after delivery, and these elevated levels can persist for up to 12–22 years. To our knowledge, no other studies evaluated IL-6 levels for a period longer than 22 years postpartum. High TNF-α levels have also been linked to the development of cardiovascular disease. As IL-6 and TNF-α play a role in cardiovascular disease pathogenesis, they might be useful as therapeutic targets. MgSO_4_ and nifedipine reduced the circulating levels of IL-6 and TNF-α in preeclamptic women [59,60]. Other studies found that IL-6 levels can be reduced by methotrexate, anakinra, and canakinumab, but none of them have been conducted on women with a history of HDP [61,62,63]. 

Laura Ormesher et al., examined echocardiographic measurements of women with a history of preterm preeclampsia treated with enalapril for six months postpartum [64]. The study found no difference between the case and control groups at six months postpartum [64]. However, women treated with enalapril had better diastolic function and left ventricular remodeling [64]. These findings suggest that enalapril treatment may reduce the long-term risk of cardiovascular disease.

According to these findings, cytokines can be used as biomarkers to predict and manage preeclampsia in its early stages. Echocardiographic changes can also indicate cardiovascular disease later in life. However, just using echocardiography to assess cardiovascular risk during pregnancy or after delivery is not advisable.

The small number of included studies limited our review. Because of the lack of studies and high variation of the biomarkers measurement’s timing, it was impossible to make a meta-analysis. To evaluate the cardiovascular risk in women, it is necessary to conduct more studies that examine changes in cytokines, angiogenic factors, and echocardiographic parameters during pregnancy and postpartum.

## Figures and Tables

**Table 1:** Postpartum changes of cytokines and angiogenic factors in women with a history of hypertensive disorder of pregnancy compared to the normotensive postpartum controls.

	TNF-α	IL-6	IL-6/IL-10	PlGF	sFlt-1	sFlt-1/PlGF
6 weeks				UC [13]: 89/5 UC [20]: 29/53	UC [20]: 29/53	UC [20]
1–3 months					UC [21]: 43/50	
3 months	↑ [22]: 17/16 UC [10]: 22/88	↑ [10,22]		↑ [23]: 23/79	UC [23]	UC [23]
9 months	UC [10]	↑ [10]				
1 year	↑ [11]: 31/40	UC [11]		UC [24]: 48/58	↑ [24]	↑ [24]
5–8 years				UC [25]: 26/15	↑ [25]	
9–16 years	UC [26]: 131/56	↑ [26]				
10 years				UC [27]: 16/18	UC [27]	
11 years				UC [12]: 15/16	UC [12]	
12 years				UC [28]: 43/21	UC [28]	UC [28]
12–22 years		↑ [29]: 249/2241				
20 years		UC [30]	↑ [30]			

TNF-α = tumor necrosis factor alpha; IL-6 = interleukin-6; IL-6/IL-10 = interleukin-6/interleukin-10 ratio; PlGF = placental growth factor; sFlt-1 = soluble fms-like tyrosine kinase-1; UC = unchanged; ↑—elevated; numbers in brackets correspond to the article number in the reference list; numbers after the colon represent case patients number/control patients number.

**Table 2:** Postpartum correlation between angiogenic factors and carotid artery intima-media parameters.

	Intima Thickness	Intima: Media Thickness Ratio (I/M)	Intima–Media Thickness (cIMT)
sFlt-1	+0.38 [24]: 48		+0.27 [28]: 43
PlGF	−0.21 [24]	−0.21 [24]	−0.25 [28]
sFlt-1/PlGF	+0.48 [24]	+0.41 [24]	

PlGF = placental growth factor; sFlt-1 = soluble fms like tyrosine kinase-1; + = positive correlation with coefficient; − = negative correlation with coefficient; Numbers in brackets correspond to the article number in the reference list; number after the colon represent case patients (if missing—it was not provided in the article).

**Table 3:** Correlation between angiogenic factors and echocardiography parameters.

	PlGF	sFlt-1
Left ventricle posterior wall thickness		+0.37 [28]: 43
Global longitudinal strain	−0.58 [28]	+0.44 [21,43]: 43
End systolic elastance		+0.27 [21]
Arterial elastance		+0.24 [21]
E/E‘		+0.096 [21]
E/A		−0.23 [21]
Aortic root diameter	− [14]: 3797	
Left atrial diameter	− [14]	
Left ventricular mass	− [14]	+0.2 [43]
Left ventricular ejection fraction		UC [43]

PlGF = placental growth factor; sFlt-1 = soluble fms like tyrosine kinase-1; UC = unchanged. + = positive correlation with coefficient (if missing—it was not provided in the article); − = negative correlation with coefficient (if missing—it was not provided in the article). Numbers in brackets correspond to the article number in the reference list; number after the colon represent case patients (if missing—it was not provided in the article).

**Table 4:** Correlation between other CVD risk factors and angiogenic, inflammatory markers.

	PlGF	sFlt-1	sFlt-1/PlGF	IL-6
Systolic blood pressure	[14]: 2924			
Diastolic blood pressure	−0.29 [28]: 43			
Mean arterial pressure	−0.25 [28]	+ [43]: 62		
Waist circumference				+0.26 [26]: 131
BMI		N [43]		+0.27 [26]
Triglycerides				+0.155 [26]
HDL	+0.34 [28]	−0.37 [28]	−0.31 [28]	−0.199 [26]

BMI = body mass index; HDL = high density lipoproteins. + = positive correlation with coefficient (if missing—it was not provided in the article) − = negative correlation with coefficient (if missing—it was not provided in the article). N—no correlation. Numbers in brackets correspond to the article number in the reference list; number after the colon represent case patients (if missing—it was not provided in the article).

## Data Availability

Not applicable.
